# Investigation of Intracellular Clearing of *Streptococcus pneumoniae* by mRNA-Encoded Cpl-1 Bacteriophage Endolysin in Human Macrophages

**DOI:** 10.3390/microorganisms14061342

**Published:** 2026-06-15

**Authors:** Carolin Warnke, Wendy Bergmann-Ewert, Marc Benjamin Janssen, Hendrik Feit Mueck, Nicolas Raether, Nooshin Mohebali, Bernd Kreikemeyer, Katharina Ekat, Moritz K. Jansson

**Affiliations:** 1 Institute of Medical Microbiology, Virology and Hygiene, University Medicine Rostock, 18057 Rostock, Germany; 2 Core Facility for Cell Sorting and Cell Analysis, University Medical Center Rostock, 18057 Rostock, Germany; 3 National and World Health Organization Supranational Reference Laboratory for Mycobacteria, Research Center Borstel, Parkallee 18, 23845 Borstel, Germany

**Keywords:** delivery strategies, mRNA therapy, bacterial infection, *Streptococcus pneumoniae*, bacteriophage endolysins, Cpl-1 endolysin, mRNA-based antibacterial therapy, mRNA-based endolysins, mRNA-encoded endolysins, intracellular killing

## Abstract

*Streptococcus pneumoniae* remains a major global health threat and is listed by the World Health Organization as a pathogen in urgent need of new antimicrobial strategies. While primarily considered an extracellular pathogen, *S. pneumoniae* can persist within splenic macrophages in severe disease, creating a protected intracellular niche that may contribute to fulminant sepsis. We recently demonstrated the concept of an mRNA-based therapeutic approach in which host cells produce the pneumococcal bacteriophage endolysin Cpl-1. Here, we investigated whether expression of Cpl-1 in macrophages can target *S. pneumoniae* residing within host cells. Using the human THP-1 macrophage line, we demonstrated successful translation and intracellular accumulation of bioactive Cpl-1 following IVT-mRNA transfection. Lysates from Cpl-1 mRNA-transfected cells exhibited bacteriolytic activity, and Western blotting as well as immunofluorescent staining confirmed cytosolic endolysin production. Phagocytosis assays using an encapsulated and unencapsulated pneumococcal strain showed a reduction in intracellular bacterial burden in Cpl-1 mRNA-transfected macrophages compared with control and inactive-mutant Cpl-1 mRNA groups, and a flow cytometry-based assay further corroborated a decreased intracellular bacterial signal. Together, these findings suggest that mRNA-encoded Cpl-1 enhances intracellular killing of *S. pneumoniae* and supports the feasibility of mRNA-based endolysin therapies to target intracellular pneumococcal reservoirs.

## 1. Introduction

*Streptococcus pneumoniae* is a leading cause of upper and lower respiratory infections such as sinusitis, otitis media, and pneumonia [1]. Despite advances in primary prevention, the global health burden of this Gram-positive encapsulated pathogen remains high. Accordingly, *S. pneumoniae* was included on the 2017 and 2024 World Health Organization priority pathogen lists of bacteria for which new antibiotics are urgently needed [2,3]. Resistance against small molecule drugs have resulted in the investigation of innovative therapeutic strategies, such as the use of bacteriophage endolysins, which enzymatically break down the bacterial cell wall with high specificity. Despite promising results, the wider use of bacteriophage endolysins is still limited mainly by challenges related to their delivery to infection sites in the human body. Intracellular targeting of microorganisms using exogenously applied bacteriophage endolysins has been successfully investigated but relies on modifications such as fusion with cell-penetrating peptides [4].

Recently, we have proposed a novel approach to the treatment of *S. pneumoniae* based on the delivery of in vitro transcribed mRNA (IVT-mRNA) encoding for the bacteriophage endolysin Cpl-1, derived from the Cp-1 bacteriophage, thereby enabling the recipient organism to produce the endolysin endogenously. mRNA-based production and cytosolic accumulation of bioactive Cpl-1 endolysin was successfully demonstrated in three human cell lines. As *S. pneumoniae* is considered to be a primarily extracellular pathogen, a secretory variant termed hlySP-sCpl-1N215D was developed that allowed for accumulation of the endolysin in cell culture supernatants [5].

However, it has been shown that in severe forms of pneumococcal disease, splenic macrophages provide a reservoir for *S. pneumoniae* constituting an intracellular niche that requires targeted treatment strategies to prevent pneumococcal sepsis [6,7].

Although our recent study did not provide proof of intracellular killing, we hypothesized that an mRNA-based therapy approach might be suitable for targeting intracellular pathogens, since mRNA therapies rely on the delivery of their nucleic acid payload to the cytosol by design [8]. Transfection and consecutive Cpl-1 production inside cells might thus facilitate intracellular targeting of *S. pneumoniae*. As a clinical application, Cpl-1 encoding mRNA delivered to splenic macrophages could potentially clear intracellular *S. pneumoniae* to prevent fulminant forms of pneumococcal sepsis. However, the efficiency of intracellular Cpl-1 could be influenced by the intracellular lifestyle of the pneumococcus such as residence within vacuoles [9].

In this study we investigated the feasibility of mRNA as a delivery strategy for the Cpl-1 bacteriophage endolysin to target *S. pneumoniae* inside human macrophages. As a first step, we examined the production of mRNA-encoded Cpl-1 in the human macrophage THP-1 cell line. In a second step a macrophage infection model was used to test the intracellular lytic activity of mRNA-encoded Cpl-1 endolysin against *S. pneumoniae*.

## 2. Materials and Methods

### 2.1. mRNA Preparation

mRNA was prepared as previously described [5]. Briefly, the Cpl-1 nucleotide sequence was codon-optimized for expression in human cells (Azenta, Chelmsford, MA, USA). The nucleotide sequence of inactive Cpl-1 (iCpl-1), which was used as a control, contains two point mutations leading to the substitutions D9K and E94A, which have been shown to lead to the loss of enzymatic activity [10,11]. DNA templates containing the human alpha globin 5′ and 3′ UTRs flanking the Cpl-1 gene were synthesized and cloned into a vector (pTwist Amp High Copy) by Twist Bioscience (South San Francisco, CA, USA). Templates were PCR-amplified, incorporating a 120 bp poly(T) tail via the reverse primer to generate a poly(A) tail. Purified dsDNA templates were used for in vitro transcription (IVT) with the HiScribe T7 mRNA Kit and CleanCap AG (New England Biolabs, Ipswich, MA, USA) to produce capped mRNA under complete substitution of uridine with N1-methylpseudouridine. DNA was removed with DNase I, and RNA was purified (Monarch RNA Cleanup Kit, New England Biolabs, Ipswich, MA, USA), quantified, and adjusted to 1000 ng/μL. Quality was verified via gel electrophoresis, and aliquots were stored at −80 °C.

### 2.2. Human Cell Lines

THP-1 cells derived from DSMZ (ACC16) were maintained at 37 °C in a humidified atmosphere containing 5% CO_2_. Suspension cells were cultured in 75 cm^2^ culture flasks (Greiner Bio-One, Kremsmuenster, Austria) with 20 mL RPMI 1640 GlutaMAX supplemented with 10% fetal calf serum (FCS, Sigma Aldrich, Taufkirchen, Germany) and were passaged twice weekly. The cell density was kept between 2 × 10^5^ and 8 × 10^5^ cells/mL. For routine passaging, 16 mL of the culture was removed and discarded (ratio of 1:5). The remaining cell suspension was replenished with fresh complete medium to restore the initial culture volume.

For differentiation, THP-1 cells were adjusted to a concentration of 3 × 10^5^ cells/mL. Subsequently, the cell suspension was seeded into the required number of wells of a 24-well plate at 1 mL/well and treated with phorbol 12-myristate 13-acetate (PMA, Sigma Aldrich, Taufkirchen, Germany) at a final concentration of 100 ng/mL. After incubation for 24 h, the culture supernatant was removed, and each well was washed twice with 1 mL complete medium to remove non-differentiated cells. The wells were then replenished with 1 mL of fresh complete medium and incubated for an additional 48 h at 37 °C and 5% CO_2_ to allow macrophage maturation.

### 2.3. Transfection of Cell Lines

Transfection experiments with THP-1 macrophages were conducted in duplicate using a 24-well plate format (Greiner Bio-One, Kremsmuenster, Austria), samples were combined at collection. mRNA (500 ng per well) was mixed with Lipofectamine MessengerMAX (Thermo Fisher Scientific, Darmstadt, Germany) (1.5 μL per well) in Opti-MEM reduced serum medium (Thermo Fisher Scientific) to form mRNA–lipid complexes according to the manufacturer’s instructions. mRNA–lipid complexes were then added to the cells. Phosphate-buffered saline (PBS) was used as a transfection control. Success of mRNA transfection was ascertained by using eGFP mRNA as a positive control.

### 2.4. Processing of Transfected Cells

Then, 24 h post-transfection, culture supernatants were removed from well plates. Cells were harvested using 200 µL TrypLE (Thermo Fisher Scientific) per well, and duplicates were combined in 2 mL tubes. Wells were then washed using 200 μL PBS. Cells were subjected to osmolysis using 81 μL of distilled water and three subsequent freeze–thaw cycles. Lysis was stopped by the addition of 9 μL 10-fold concentrated PBS followed by centrifugation at 16,000× *g* for 15 min (4 °C). The cell lysate (approx. 90–100 μL) was transferred to 1.5 mL tubes and used for subsequent analysis.

### 2.5. Western Blot Analysis

For SDS-PAGE analysis, cell lysates were separated on 12% polyacrylamide gels. Following SDS-PAGE, separated proteins were transferred onto nitrocellulose membranes. Western blot analysis was performed by applying a polyclonal anti-Cpl-1 antibody (Davids Biotechnologie GmbH, Regensburg, Germany) overnight (1:1000). Blots were incubated with a secondary fluorophore-labeled antibody (IRDye^®^ 680RD Goat anti-Rabbit IgG, LI-COR Biosciences—GmbH, Bad Homburg, Germany) for 1 h (1:10,000) and imaged using an Odyssey Imager (LI-COR Biosciences).

### 2.6. Immunocytochemistry Analysis

THP-1 cells were transferred to 24-well plates with glass coverslips (Carl Roth, Karlsruhe, Germany); differentiation and mRNA transfection were conducted as described above. Next, 24 h after transfection, cells were fixed using 4% paraformaldehyde and then permeabilized using 0.5% PBST for 10 min, followed by blocking using PBS containing 5% FCS. Cells were incubated with a polyclonal anti-Cpl-1 antibody (Davids Biotechnologie) for 2–3 h (1:1000). After washing thoroughly with PBS, a conjugated secondary antibody (Goat anti-Rabbit IgG [H + L], DyLight 594, Thermo Fisher Scientific) was applied for 2–3 h (1:100). After washing with PBS, DAPI (300 nM, Thermo Fisher Scientific) was applied for 5 min. Coverslips were treated with mounting medium (ROTIMount FluorCare, Carl Roth GmbH, Karlsruhe, Germany) then placed on a microscope slide. Slides were analyzed using an Olympus BX60 fluorescence microscope (Olympus, Tokyo, Japan), and images were acquired using an RT Color CCD Camera (Diagnostic Instruments Inc, Sterling Heights, MI, USA).

### 2.7. Handling of S. pneumoniae and CDFA-SE Staining

Unencapsulated *S. pneumoniae* strain R6 and capsulated strains TIGR4 (serotype 4) and ATCC 49619 (serotype 19F) were grown in THY medium. For R6 and TIGR4, stocks were produced at a concentration of 2.5 × 10^8^ CFU/mL and labeled using CDFA-SE (Life Technologies, Waltham, MA, USA) according to the manufacturer’s instructions. In short, a 20 mM working solution of CFDA-SE was mixed with the bacterial suspension at equal volumes and incubated for 30 min at 37 °C in the dark. Following incubation, the suspension was washed three times with PBS and centrifuged at 4000× *g* for 5 min after each wash. The final pellet was resuspended in the original volume of PBS containing 10% glycerol, stocks were stored at –80 °C until use.

### 2.8. Turbidity Reduction Assay and CFU Determination

To assess biological activity of mRNA-based Cpl-1 endolysin, turbidity reduction measurements were conducted with cell lysates of transfected cells in a 96-well format using a Spectramax M2 microplate reader device (Molecular Devices, San Jose, CA, USA). Then, 22 µL of cell lysates from transfected THP-1 macrophages were added to 96-well plates in triplicate including non-transfected control lysates. *S. pneumoniae* (ATCC 49619) was cultured in THY medium to logarithmic phase, reaching an OD_600_ of approx. 0.4. Bacteria were then washed and resuspended in PBS (pH: 7.0) to an OD_600_ of 0.9 using a spectrophotometer (DeNovix, Wilmington, NC, USA). Next, 200 µL of *S. pneumoniae* in PBS (pH: 7.0) was added to transfected/non-transfected THP-1 macrophage cell lysates in 96-well plates. Pneumococcal solution was combined with recombinantly expressed Cpl-1 endolysin (final concentration of 5 μg/mL) as a positive control. Turbidity at OD_600_ was measured at 37 °C every minute for 60 min. After the experiment, samples and controls were plated on blood agar following serial 10-fold dilutions. CFUs were determined after overnight incubation at 37 °C in a 5% CO_2_ atmosphere.

### 2.9. Phagocytosis and Extracellular Killing Assay

Bacterial suspensions were prepared by mixing 250 µL of bacteria (2.5 × 10^8^ CFU/mL) with 250 µL of 20% pooled human serum in HBSS and incubating the mixture for 20 min at 37 °C under gentle agitation for opsonization. THP-1 macrophages (untransfected, iCpl-1 mRNA-transfected, Cpl-1 mRNA-transfected) were detached with TrypLE, washed with HBSS and adjusted to approx. 1.0 × 10^6^ cells/mL. For infection, 80 µL of opsonized bacteria (MOI approx. 125) were added to the cells, followed by incubation for 30 min at 37 °C under gentle shaking. Cells were then washed twice with HBSS via centrifugation at 400× *g* to remove extracellular bacteria. To eliminate remaining extracellular bacteria, cells were incubated for 30 min in RPMI containing 400 µg/mL gentamicin (Gibco^®^ Life Technologies, Darmstadt, Germany) and 100 U/mL penicillin G (Carl Roth GmbH + Co. KG, Karlsruhe, Germany). After centrifugation and washing with HBSS, intracellular bacteria were released by adding 500 µL of 2.5% saponin (pH 9). Lysates and processed supernatants were plated in serial dilutions on agar plates, and CFUs were quantified after an overnight incubation.

### 2.10. Flow Cytometry

For each bacterial strain, 400 µL of CFDA-SE-labeled bacterial stocks (2.5 × 10^8^ CFU/mL in HBSS) were mixed with 400 µL of 20% human serum in HBSS and incubated for 20 min at 37 °C under gentle shaking for opsonization. THP-1 macrophages (untransfected, iCpl-1 mRNA-, Cpl-1 mRNA-transfected) were detached using TrypLE, washed with HBSS, centrifuged at 400× *g* for 5 min, and resuspended in HBSS to a density of approx. 1 × 10^6^ cells/mL. For infection, 200 µL of THP-1 macrophage suspension was combined with 160 µL of the opsonized bacteria (MOI approx. 100) and incubated for 30 min at 37 °C under gentle agitation. Cells were then centrifuged at 400× *g* for 5 min and washed twice with 2 mM EDTA in PBS (pH 7.4) to remove extracellular bacteria.

For flow cytometric staining, cell pellets were resuspended in 100 µL PBS + EDTA. Cell viability was assessed using the Zombie NIR kit (BioLegend, Dan Diego, CA, USA) by adding the dye at a final 1:2000 dilution, followed by incubation for 15–30 min at room temperature in the dark. Cells were washed twice with 1 mL of PBS + EDTA. Trypan blue was used to quench the signal of THP-1-associated but not internalized *S. pneumoniae*. For trypan blue quenching, samples were resuspended in 250 µL PBS+ EDTA and incubated with 1.2 mg/mL trypan blue (Gibco^®^ Life Technologies, Darmstadt, Germany) for 5 min before being washed twice with PBS+ EDTA and resuspended in 80 µL PBS + EDTA.

For surface antibody staining, cells were incubated with 5 µL Monocyte Blocker and 5 µL TruStain FcX (BioLegend) for 5–10 min at room temperature, followed by staining with APC anti-human CD11b (BioLegend) (1.5 µg/mL) for 10–15 min. Cells were washed with at least 1 mL PBS + EDTA and fixed in 200 µL of 4% paraformaldehyde for 10 min. Cells were washed twice in Perm Wash Buffer (BioLegend) and finally resuspended in PBS + EDTA for analysis. For data acquisition, a BD FACSVerse (BD Biosciences, Franklin Lakes, NJ, USA) equipped with a blue and red laser and BD FACSuite™ software version 1.0.6. was used.

The gating strategy proceeded as follows: (1) identification of the main THP-1 macrophage population using CD11b vs. SSC-H; (2) exclusion of dead cells based on Zombie NIR-staining to gate live cells; (3) selection of single cells using SSC-A vs. SSC-H; and (4) assessment of FITC signal, indicating intracellular presence of CFDA-SE-labeled *S. pneumoniae*.

### 2.11. Data Analysis and Statistics

Flow cytometry data were visualized, plotted and gated using FlowJo v10. Data were analyzed and visualized using GraphPad Prism 10 (GraphPad Software, Boston, MA, USA). Samples for each experiment were run in technical triplicate, and all experiments were repeated at least three times to account for natural biological variation. For comparison of absolute CFU counts between multiple groups in turbidity reduction experiments Welch’s ANOVA was used under assumption of unequal variance followed by Dunnett’s T3 multiple comparison test to compare against a single control group. In phagocytosis assays and flow cytometry experiments mean values were expressed as percentages of control values. Comparisons between groups were performed using a two-tailed *t*-test under assumption of equal variances. Results are based on at least three independent experiments. *p* values < 0.05 were considered significant.

## 3. Results

### 3.1. Cpl-1-Encoding mRNA Was Translated into Bioactive Cpl-1 Endolysin in THP-1 Macrophages

**Figure 1:**
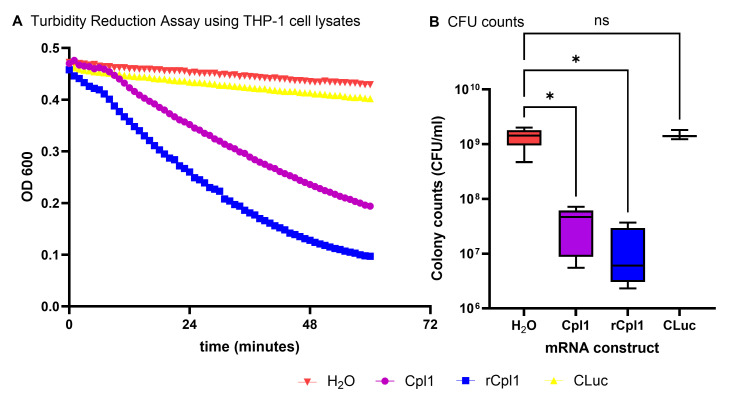
Bacteriolytic effect of lysate samples from THP-1 cells transfected with Cpl-1-encoding mRNA against *S. pneumoniae*. (**A**) Turbidity reduction experiments. First, 22 µL of lysates of cells were transfected with mRNA encoding for Cpl-1 endolysin (Cpl1), then Cypridina luciferase (CLuc) or mock-transfected cells (H_2_O) were applied to 200 μL of pneumococcal suspension in PBS (pH 7.0), and OD_600_ was measured every minute for 60 min. Recombinantly expressed Cpl-1 at 5 µg/mL was used as positive control (rCpl1). Data from representative experiments are shown. (**B**) After turbidity measurements, samples were plated on blood agar and CFUs were counted after 16 h. Data are based on at least three independent experiments. Boxplots represent minimum, maximum, and median values, and the interquartile range. *p* values < 0.05 are indicated (* *p* < 0.05), ns: not significant.

THP-1 cells were differentiated in 24-well plates using 100 ng/mL PMA, followed by transfection using 500 ng Cpl-1 encoding mRNA. Then, 24 h after transfection, THP-1 cells were lysed; 22 µL of lysates were added to 200 µL of pneumococcal suspensions and turbidity (OD_600_) was observed over time (Figure 1A). After 60 min, the colony counts of bacterial suspensions treated with lysates of Cpl-1 encoding mRNA-transfected cells were significantly lower (*p* < 0.01) when compared with samples treated with control lysates (H_2_O), showing a log reduction of 1.56 (Figure 1B). This was slightly lower but comparable to the effect observed using 5 µg/mL of recombinantly produced Cpl-1 (rCpl-1, log reduction of 2.03). To account for mRNA-induced non-specific effects on bacterial viability, lysates of THP-1 cells transfected with mRNA encoding for Cypridina Luciferase (CLuc) were used as an additional control and no noticeable CFU reduction was observed (*p* = 0.961) (Figure 1B).

**Figure 2:**
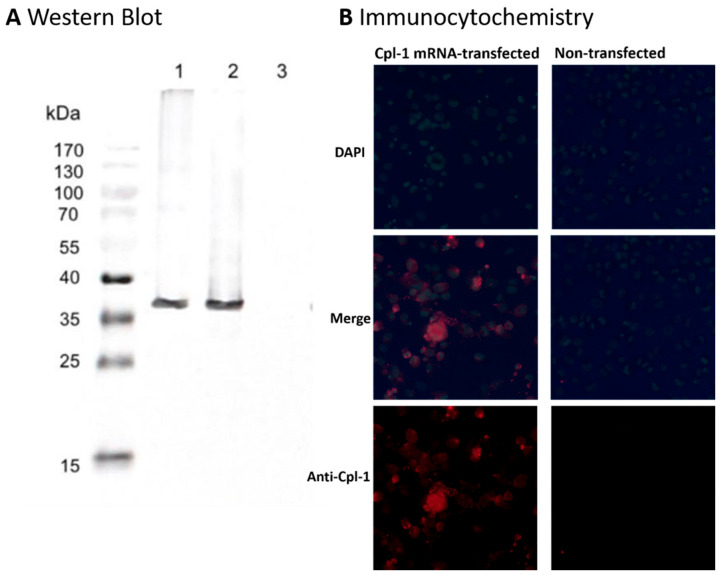
Western blot of lysate samples of THP-1 cells and immunofluorescent staining. (**A**) First, 10 µL of cell lysates of THP-1 cells harvested 24 h after transfection were subjected to Western blot analysis. 1: recombinant Cpl-1 (0.4 µg, approx. 37 kDa). 2: Cpl-1-encoding mRNA 3: non-transfected control lysate. (**B**) THP-1 cells were stained using an anti-Cpl-1 rabbit-derived antibody and conjugated goat anti-rabbit secondary antibody; DAPI was used for staining of cell nuclei. The panels show DAPI (blue), DAPI + Anti-Cpl-1 (blue/red), and Anti-Cpl-1-staining (red) of Cpl-1 mRNA-transfected and non-transfected cells, respectively. Pictures were taken at ×200 magnification.

When subjected to Western blot, a prominent band with a comparable molecular weight to rCpl-1 was observed exclusively in lysate samples of THP-1 cells that had been transfected with mRNA coding for the Cpl-1 endolysin, indicating the presence of the Cpl-1 endolysin in the transfected cells (Figure 2A). The results of the turbidity reduction assay with successive CFU quantification and Western blot imaging suggest that mRNA encoding for Cpl-1 endolysin leads to the production and intracellular accumulation of the bioactive Cpl-1 endolysin in THP-1 macrophages. Immunofluorescent staining of transfected THP-1 cells suggested cytosolic presence of Cpl-1 (Figure 2B).

### 3.2. A Phagocytosis Assay Showed Reduced Survival of S. pneumoniae Inside THP-1 Macrophages Transfected with Cpl-1-Encoding mRNA

**Figure 3:**
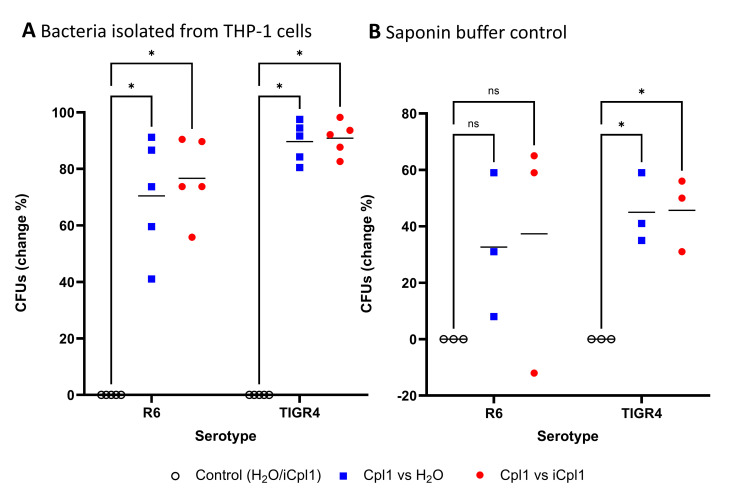
Effect of Cpl-1 mRNA on survival of R6/TIGR4 strains of *S. pneumoniae* inside THP-1 cells. (**A**) THP-1 cells were transfected using Cpl-1 mRNA (Cpl1), iCpl-1 mRNA (iCpl1), or water controls (H_2_O). CFU counts of *S. pneumoniae* were determined from cell lysates after phagocytosis by THP-1 cells and compared with the other groups. (**B**) To exclude the possibility of post-lysis killing of phagocytosed *S. pneumoniae* due to release of Cpl-1 endolysin from THP-1 cells, saponin lysis buffer at pH 9 was spiked with R6/TIGR4 pneumococci and used for cell lysis of uninfected THP-1 cells. Data are based on at least three independent experiments. *p* values <0.05 are considered significant (* *p* < 0.05), ns: not significant.

As a next step, the effect of Cpl-1 mRNA on intracellular survival of *S. pneumoniae* inside THP-1 macrophages was investigated. To this end, transfected and non-transfected THP-1 cells were subjected to infection by *S. pneumoniae* using a phagocytosis assay in conjunction with a protection assay with gentamicin and penicillin to eliminate extracellular pneumococci. The non-encapsulated strain R6 and encapsulated strain TIGR4 were included to test for the effect of the pneumococcal capsule on Cpl-1-mediated intracellular killing. mRNA encoding for an inactive form of Cpl-1 (iCpl-1) was used to control for non-specific effects of mRNA transfection on intracellular survival of *S. pneumoniae*. We considered it an important caveat that Cpl-1 and *S. pneumoniae* released during cell lysis could mix in the lysis buffer, leading to bacterial killing after lysis rather than within the THP-1 macrophages. To account for this, the saponin lysis buffer was adjusted to pH 9.0, a range where the Cpl-1 endolysin shows negligible activity [12]. Following phagocytosis THP-1 cells were lysed and CFUs were determined. Cell lysates of THP-1 cells transfected with mRNA encoding for Cpl-1 showed significantly lower CFUs compared to non-transfected (H_2_O) and iCpl-1 mRNA-transfected cells for both serotypes (R6: Cpl-1 vs. H_2_O *p* = 0.002, Cpl-1 vs. iCpl-1 *p* < 0.001; TIGR: Cpl-1 vs. H_2_O *p* < 0.001, Cpl-1 vs. iCpl-1 *p* < 0.001) (Figure 3A). To determine whether bacterial killing occurred extracellularly through contact with Cpl-1 released during lysis of mRNA-transfected cells, the experiments were repeated under modified conditions. Instead of performing a phagocytosis assay, bacteria were directly added to saponin buffer (pH 9) prior to lysis of THP-1 cells. CFUs recovered from the saponin buffer of Cpl-1 mRNA-transfected cells after lysis were lower than those obtained from iCpl-1 mRNA and H_2_O controls. However, the difference was less pronounced than the CFU reductions observed in the phagocytosis assay (R6: Cpl-1 vs. H_2_O *p* = 0.157, Cpl-1 vs. iCpl-1 *p* = 0.270; TIGR: Cpl-1 vs. H_2_O *p* = 0.047, Cpl-1 vs. iCpl-1 *p* = 0.026; One sample *t*-test) (Figure 3B). The results suggest that while some bacterial killing might occur in the saponin buffer because of lysis-mediated mixing of Cpl-1 and *S. pneumoniae*, an independent killing effect takes place inside the Cpl-1 mRNA-transfected macrophages.

### 3.3. A Flow Cytometry-Based Assay Was Established and Showed Reduced Intracellular Burden of S. pneumoniae Inside Macrophages Transfected with Cpl-1-Encoding mRNA

**Figure 4:**
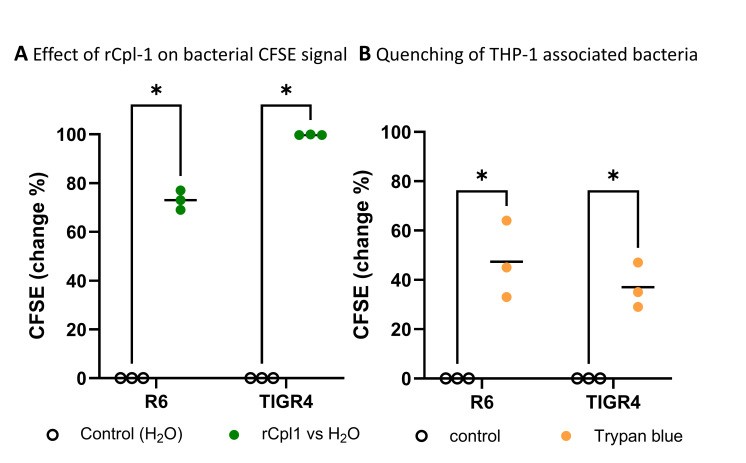
Flow cytometry analysis of the effect of rCpl-1 and trypan blue on bacterial CFSE signal. (**A**) Treatment with recombinant Cpl-1 (rCpl-1) causes a significant reduction in CFSE fluorescence in CFDA-SE-labeled *S. pneumoniae* R6 and TIGR4 strains, indicating that a reduction in bacterial CFSE signal can be used to determine Cpl-1-mediated bacterial lysis. (**B**) Trypan blue treatment affects CFSE signal of THP-1 cells incubated with CFDA-SE-labeled *S. pneumoniae* R6 and TIGR4 strains, suggesting quenching of THP-1 cell-associated but not internalized bacteria. Data are presented as mean from at least three independent experiments; each dot represents an individual replicate. Statistical analysis was performed using one sample *t*-tests; *p* < 0.01 considered significant (* *p* < 0.05).

A flow cytometry-based assay using CFDA-SE-labeled *S. pneumoniae* was established to further investigate the effect of Cpl-1 mRNA on bacteria residing inside THP-1 macrophages. In a first step we determined whether Cpl-1 treatment of CFDA-SE-labeled *S. pneumoniae* results in a measurable reduction in CFSE signal. CFDA-SE-labeled R6 and TIGR4 bacteria were incubated with 10 µg/mL recombinantly produced Cpl-1 and the CFSE signal was successively measured. In comparison with non-Cpl-1 treated samples, the CFSE signal was reduced by 73% for R6 (*p* < 0.001) and 99.8% for TIGR4 (*p* < 0.001), respectively (Figure 4A). Our interest was to determine the fate of intracellular bacteria in Cpl-1 mRNA-transfected THP-1 macrophages. To differentiate between intracellular bacteria and cell membrane-associated but not-phagocytosed bacteria, trypan blue quenching was used as previously described in other studies [13,14]. To investigate the effect of trypan blue on *S. pneumoniae* adherent to THP-1 macrophages following phagocytosis assay, the CFSE signal was measured and compared to untreated THP-1 macrophages. Trypan blue treatment led to a moderate reduction in CFSE signal of 47.33% for R6 (*p* = 0.035) and 37% for TIGR4 (*p* = 0.020) (Figure 4B).

**Figure 5:**
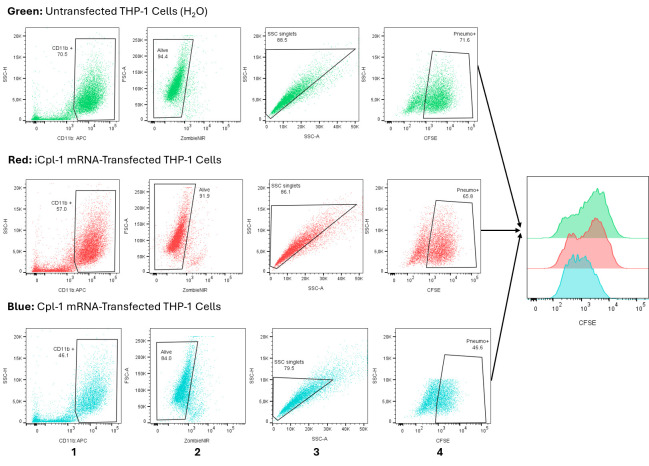
Flow cytometry gating strategy for analysis of THP-1 cells. Infected THP-1 cells were analyzed across three experimental groups: untreated control (H_2_O), Cpl-1 mRNA-treated, and iCpl-1 mRNA treated. Gating strategy: (1) identification of the main THP-1 population using CD11b vs. SSC-H; (2) exclusion of dead cells based on Zombie NIR-staining to gate live cells; (3) selection of single cells using SSC-A vs. SSC-H; and (4) assessment of CFSE signal, indicating intracellular presence of CFDA-SE-labeled *S. pneumoniae*. Histograms show the distribution of CFSE fluorescence in each group.

Using the approach described above, the final flow cytometry assay analyzed THP-1 cells across three experimental groups: untreated control (H_2_O), Cpl-1 mRNA-treated, and iCpl-1 mRNA-treated. THP-1 macrophage differentiation was assessed using CD11b, viability was assessed based on Zombie NIR- staining. The gating strategy is outlined in Figure 5.

**Figure 6:**
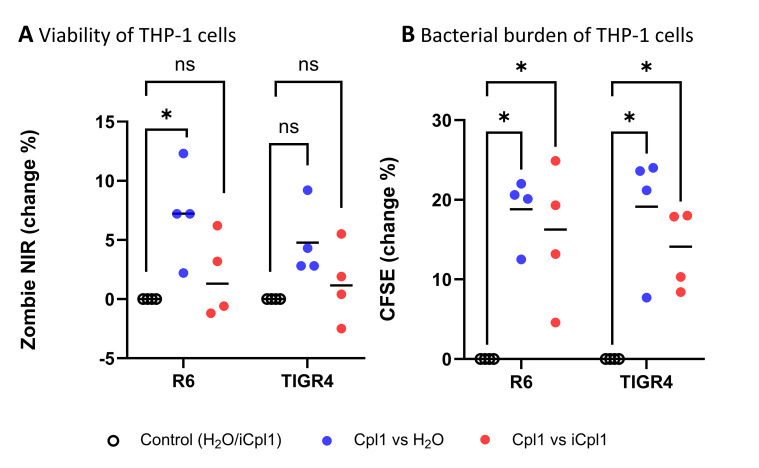
Flow cytometry analysis of THP-1 cell viability and intracellular bacterial burden. (**A**) THP-1 cell viability, measured by Zombie NIR staining following transfection with mRNA encoding for Cpl-1 (Cpl1) or its inactive variant iCpl-1 (iCpl1) compared to control transfection (H_2_O). (**B**) Phagocytosis assay showing change in CFSE fluorescence in THP-1 cells transfected with Cpl-1 mRNA relative to iCpl-1 mRNA/H_2_O control after exposure to CFDA-SE-labeled R6 and TIGR4 bacteria. Data are presented as the mean from at least three independent experiments; each dot represents an individual replicate. Statistical analysis was performed using one-sample *t*-tests; *p* < 0.01 considered significant (* *p* < 0.05), ns: not significant.

Viability of Cpl-1 mRNA-transfected THP-1 cells was compared to sham transfected cells (H_2_O) and iCpl-1 mRNA-transfected cells using Zombie NIR signal. For cells infected with R6, Cpl-1 mRNA transfection was associated with an increased cell death of 7.23% compared to H_2_O (*p* = 0.040) and 1.90% compared to iCpl-1 (*p* = 0.350). For cells infected with TIGR4, Cpl-1 mRNA transfection was associated with an increase in cell death of 4.78% compared to H_2_O (*p* = 0.051) and 1.33% compared to iCpl-1 (*p* = 0.484). Internal residence of CFDA-SE-labeled R6 and TIGR4 bacteria inside THP-1 cells was assessed by CFSE intensity. For R6-infected THP-1 cells, a mean reduction in CFSE signal of 18.80% was observed in Cpl-1 mRNA-transfected cells compared to H_2_O (*p* = 0.003) and 15.50% compared to iCpl-1 (*p* = 0.038). For TIGR4-infected THP-1 cells, a mean reduction in CFSE signal of 19.13% was observed in Cpl-1 mRNA-transfected cells compared to H_2_O (*p* = 0.016) and 13.65% compared to iCpl-1 (*p* = 0.012) (Figure 6).

In summary, the CFSE signal was lower in Cpl-1 mRNA-transfected THP-1 cells. These findings are in line with the quantitative reduction in bacteria observed in the phagocytosis assay outlined above and suggest a lower bacterial burden inside Cpl-1 mRNA-transfected THP-1 cells. Together these results seem to indicate that Cpl-1 mRNA transfection of macrophages results in an enhanced intracellular reduction in *S. pneumoniae*.

## 4. Discussion

The intracellular environment represents an important niche for numerous bacterial pathogens, yet effective targeting of bacteria within host cells remains challenging [15]. Only a limited number of antibiotics achieve sufficient therapeutic drug levels in the cytosol of host cells. Macrolides are amongst the few known substances to effectively clear intracellular bacteria and seem to positively affect the clinical course of *S. pneumoniae*, arguably due to the capability of clearance from infected splenic macrophages [6]. Macrolide resistance in *S. pneumoniae*, first detected in 1967 in Canada, has become widespread in parts of Asia and Europe with resistance rates frequently exceeding 30–50% [16].

In this context, our study demonstrates the feasibility of using mRNA transfection to induce intracellular production of the pneumococcal bacteriophage endolysin Cpl-1 in a human macrophage cell line. Transfected macrophages remained largely viable in infection experiments, and both quantitative CFU-based and flow-cytometry-based phagocytosis assays indicated Cpl-1-mRNA-mediated intracellular reduction in encapsulated and unencapsulated pneumococcal strains. While both assays indicate a bacterial reduction, CFU-based assays tended to show a higher relative reduction in the intracellular bacterial burden. Reasons for these observations could lie in the CFU-based detection of structurally intact but non-viable bacteria in flow cytometry that could not be detected in CFU-based assays. Another explanation could be incomplete quenching of THP-1-associated but not internalized bacteria, resulting in an underestimation of the actual reduction in intracellular bacterial load. Regarding the scope of our study, these data show that mRNA-encoded endolysins are a promising strategy to target bacteria in intracellular compartments.

The spleen, which harbors a significant population of macrophages, which have been shown to be implicated in pneumococcal persistence and replication, represents an attractive target for such an approach [6,7]. While intramuscular mRNA delivery to the human body has been transformative in vaccinology, systemic administration remains a challenge mainly due to immunogenicity elicited by nanocarriers and limited organ-specific targeting capabilities. Of note, systemic application using ionizable lipid nanoparticles (LNPs) leads to rapid accumulation in the liver due to interactions with apolipoprotein E and successive uptake by LDL receptors [17]. Recent advances in LNP-based delivery platforms—have circumvented liver tropism and enable efficient transfection of splenic antigen-presenting cells, including splenic macrophages, primarily in the context of cancer immunotherapies [17,18,19,20,21,22]. Leveraging these established delivery technologies to transport Cpl-1 mRNA specifically to the spleen could enable selective elimination of intracellular *S. pneumoniae* from inside splenic macrophages. Clinically, such a strategy may hold value in severe pneumococcal disease, where clearance of intracellular bacteria could prevent replication and subsequent bacteremia.

Although macrophage transfection has been shown previously, avoiding excessive macrophage immune stimulation remains a key hurdle due to innate immune sensing of exogenous mRNA by various endosomal and cytosolic pattern recognition receptors. Examples include Toll-like receptors 3, 7, and 8, retinoic acid inducible gene I (RIG-I) or melanoma differentiation-associated protein 5 (MDA5) [23,24]. Chemical modifications of nucleotides provide an effective strategy to mitigate immune activation. The N^1^-methylpseudouridine modification, initially described by Katalin Karikó et al., was used in our study and has been shown to reduce macrophage inflammatory response [23,24,25]. However, bacterial clearance due to macrophage activation as a non-specific effect of IVT-mRNA needed to be considered in our study. The intracellular clearance observed here is unlikely to be attributable to non-specific effects of mRNA transfection, as the inactive Cpl-1 control mRNA served as an appropriate control. Macrophage polarization and cytokine profiles in response to mRNA transfection should, however, be further investigated in infection models to facilitate the tuning of the inflammatory response in bacterial infections. With advances in the understanding of LNP components the delivery modality of mRNA therapies can be tuned to elicit an immune response that acts in conjunction with the nucleic acid payload to achieve a therapeutic outcome [22]. As a relevant example, Xue et al. have recently developed an anti-inflammatory ionizable lipid to deliver mRNA encoding an antimicrobial peptide fused to an IgG1 Fc fragment to successfully target bacterial pathogens in the lung while mitigating inflammation [26].

Our data provide evidence of mRNA-based Cpl-1 endolysin-mediated killing of intracellular *S. pneumoniae* but is limited by not elucidating the intracellular dynamics of Cpl-1-mediated clearance. The observed distribution pattern of Cpl-1 endolysin using fluorescent staining suggests cytosolic accumulation while the distribution in intracellular vesicles can neither be confirmed or excluded by the methods we used. In the work of MR Oggioni’s group, transmission electron microscopy showed that *S. pneumoniae* localized within the host cell cytoplasm of splenic macrophages, without evident delimitation by a vacuolar membrane [6,7]. Partial cytosolic escape of *S. pneumococci* could therefore explain the reduction but not elimination of bacterial numbers that we observed in our study; however, this needs to be further investigated. Further work is warranted to track endolysin and bacterial localization possibly using live-cell imaging and to more comprehensively assess macrophage activation in response to combined mRNA transfection and bacterial infection. Due to the low transferability of our THP-1 cell model to in vivo macrophage physiology, the results of this study are limited to in vitro proof-of-concept. Extending these studies to primary macrophages and using clinically proven LNP formulations will be essential to validate physiological relevance. Unfortunately, CD169+ splenic macrophages, which would be an ideal model, are difficult to maintain under cell culture conditions [27]. In vivo models and spleens perfused ex vivo were employed by the group of MR Oggioni to reveal the relevance of splenic macrophages in intracellular replication of the pneumococcus [6,7]. These intricate approaches represent prime models for testing the transferability of our work. Finally, the transferability of the here-described mRNA-based approach to other relevant bacterial pathogens occupying an intracellular niche such as mycobacteria should be investigated.

In conclusion, our results suggest that mRNA-encoded Cpl-1 bacteriophage endolysin can mediate intracellular clearance of *S. pneumoniae*, supporting the potential of mRNA-based bacteriophage endolysin therapies to target bacteria in challenging intracellular reservoirs.

## Data Availability

Data that support the findings of this study not presented in the manuscript are available from the corresponding author upon reasonable request.

## Abbreviations

CD	cluster of differentiation
CFDA-SE	carboxyfluorescein diacetate, succinimidyl ester
CFSE	5(6)-carboxyfluoreszeindiacetat-N-succinimidylester
CFUs	colony forming units
CLuc	Cypridina luciferase
DAPI	4′,6-diamidino-2-phenylindole
DSMZ	German Collection of Microorganisms and Cell Cultures GmbH
EDTA	ethylenediaminetetraacetic acid
FCS	fetal calf serum
HBSS	Hanks’ Balanced Salt Solution
iCpl-1	inactive Cpl-1
IVT	in vitro transcription
LNPs	lipid nanoparticles
MDA5	melanoma differentiation-associated protein 5
min	minutes
mM	millimolar
MOI	multiplicity of infection
mRNA	messenger ribonucleic acid
nM	nanomolar
OD_600_	optical density at wavelength at 600 nm
Opti-MEM	Optimized-Minimal Essential Medium
PBS	phosphate-buffered saline
PBST	phosphate-buffered saline with tween detergent
PCR	polymerase chain reaction
PMA	phorbol 12-myristate 13-acetate
rCpl-1	recombinantly produced Cpl-1
RIG-I	retinoic acid inducible gene I
RPMI medium	Roswell Park Memorial Institute medium
SDS-PAGE	sodium dodecyl sulfate polyacrylamide gel electrophoresis
SSC	side scatter
SSC-A	side scatter area
SSC-H	side scatter height
T	thymine
THY medium	Todd–Hewitt broth supplemented with yeast extract
UTR	untranslated region
WHO	World Health Organization
